# Feasibility, safety, and acceptability of a remotely monitored exercise pilot CHAMP: A Clinical trial of High‐intensity Aerobic and resistance exercise for Metastatic castrate‐resistant Prostate cancer

**DOI:** 10.1002/cam4.4324

**Published:** 2021-10-12

**Authors:** Stacey A. Kenfield, Erin L. Van Blarigan, Neil Panchal, Alexander Bang, Li Zhang, Rebecca E. Graff, Yea‐Hung Chen, Charles J. Ryan, Anthony Luke, Robert U. Newton, Imelda Tenggara, Brooke Schultz, Elizabeth Wang, Emil Lavaki, Kyle Zuniga, Nicole Pinto, Hala Borno, Rahul Aggarwal, Terence Friedlander, Vadim S. Koshkin, Andrea Harzstark, Eric Small, June M. Chan

**Affiliations:** ^1^ University of California, San Francisco San Francisco California USA; ^2^ Weill Cornell Medical College New York New York USA; ^3^ University of Minnesota Minneapolis Minnesota USA; ^4^ Edith Cowan University Perth Australia; ^5^ Columbia University New York New York USA; ^6^ UCLA Los Angeles California USA; ^7^ Kaiser Permanente Oakland Medical Center Oakland California USA

**Keywords:** behavioral intervention, physical activity, remote, strength training

## Abstract

**Background:**

Exercise may improve clinical and quality of life outcomes for men with prostate cancer. No randomized controlled trials (RCTs) have examined the feasibility, safety, and acceptability of remote exercise training in men with metastatic castrate‐resistant prostate cancer (mCRPC).

**Methods:**

We conducted a pilot RCT (1:1:1 aerobic or resistance exercise 3x/week or usual care) to determine the feasibility, safety, and acceptability of remotely monitored exercise over 12 weeks in 25 men with mCRPC. A prescribed exercise program was based on baseline testing including high‐ and moderate‐intensity aerobic exercise or resistance exercise completed at a local exercise facility. Feasibility was based on attendance, adherence, and tolerance; safety on adverse events; and acceptability on participant interviews.

**Results:**

Between March 2016 and March 2020, 25 patients were randomized (8 aerobic, 7 resistance, and 10 control). Twenty‐three men (82%) completed the 12‐week study. Men who completed the remote intervention attempted 90% and 96% of prescribed aerobic and resistance training sessions, respectively, and 86% and 88% of attempted sessions were completed as or more than prescribed. We observed changes in performance tests that corresponded with the exercise prescription. No safety concerns were identified. Ninety percent of participants interviewed were satisfied with the program and would recommend it to others.

**Conclusions:**

Remotely monitored exercise training is feasible, safe, and acceptable in men with mCRPC; there was no difference in these outcomes by mode of exercise. Through this research, we provide direction and rationale for future studies of exercise and clinical outcomes in patients with metastatic prostate cancer.

## Graphical abstract

We conducted a 3‐arm pilot randomized controlled trial to determine the feasibility, safety, and acceptability of two modes of remotely monitored exercise (resistance or aerobic) over 12 weeks compared to usual care in 25 men with metastatic castrate‐resistant prostate cancer (mCRPC). We report that remotely monitored exercise training is feasible, safe, and acceptable in this patient population, with a high satisfaction rate. Through this research we provide direction and rationale for future studies of exercise and clinical outcomes for people with metastatic cancer.

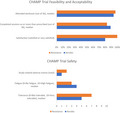

## 1 INTRODUCTION

Prostate cancer (PC) remains the second leading cause of cancer death among men in the United States (US)^1^ and the fifth leading cause of death among men worldwide.^2^ Significant advances have been made in therapies for advanced PC^3^; median survival is approximately 32 months for metastatic castrate‐resistant PC (mCRPC).^4^ However, these therapies have severe cardiovascular and metabolic effects; negatively impact cognitive, psychological, muscle, and bone health; and may lead to decreased cardiorespiratory fitness, frailty, and reduced quality of life (QOL).^5^ Adjuvant interventions, such as exercise, have promise for preventing or reducing detrimental side effects of therapy for advanced PC and warrant further examination in men with mCRPC.

The effects of aerobic and resistance exercise in men with PC have been examined in multiple randomized controlled trials (RCTs), evaluated in systematic reviews,^6^, ^7^ and reported that exercise interventions can improve the quality of life (including fatigue), fitness, and function for men with PC. For example, one study in men undergoing androgen deprivation therapy (ADT) reported that an onsite supervised multimodal (aerobic and resistance training) program significantly improved muscle mass, physical strength, walk times, and general health, and also reduced fatigue.^8^ Few studies have been completed in men with metastatic PC due to perceived elevated risk of skeletal‐related events. In a 12‐week RCT of *onsite* supervised resistance exercise (2/week) among 20 men with bone metastases, 75% completed the study. No adverse events or skeletal complications occurred during exercise, and attendance, exercise tolerance, and rated perceived exertion (RPE) were high.^9^ In a 12‐week efficacy and safety study of 57 PC patients with bone metastasis randomized to multimodal supervised exercise (3x/week) versus control, attendance, tolerance, and RPE were similar to the study by Cormie et al.^9^ and exercise increased self‐reported physical functioning and objectively measured lower body muscle strength, with no differences in adverse events or skeletal fractures.^10^ Notably, these studies focused on men with bone metastases and tested the safety and feasibility of *onsite supervised* exercise.

The feasibility, safety, and effects of *remote‐based* aerobic or resistance exercise in men with metastatic castrate‐resistant prostate cancer (mCRPC) are unknown. Thus, we conducted the CHAMP study (A **C**linical trial of **H**igh‐intensity **A**erobic and resistance exercise for **M**etastatic **P**rostate cancer) and report here on the primary (feasibility, safety, and acceptability) and secondary (cardiovascular fitness, strength, and Halabi prognostic score) outcomes.^11^

## 2 MATERIALS AND METHODS

### 2.1 Study design, recruitment, and eligibility

CHAMP was a three‐arm 12‐week pilot RCT among men with mCRPC. Main inclusion criteria included: histologically confirmed PC and clinically confirmed castration resistance; Eastern Cooperative Oncology Group (ECOG) performance status of 0–1; clearance to undergo a maximal steep ramp exercise test on a cycle ergometer and complete vigorous aerobic and resistance exercise training; and English proficiency. Main exclusion criteria included: contraindications to exercise (e.g., serious cardiovascular event within 12 months); poorly controlled hypertension; spinal cord compromise; moderate‐to‐severe bone pain; self‐reported ≥75 min/week of vigorous aerobic exercise; or ≥3 days/week of structured resistance exercise (see File S1 for all criteria). Patients reporting chest pain, medically supervised activity, or shortness of breath performing activities of daily living required cardiologist clearance. Pre‐CRPC chemotherapy was allowed; post‐CRPC chemotherapy was allowed with physician approval. Men were allowed to have past or current treatment with abiraterone or enzalutamide. All participants were required to be on ADT during the study period. Written informed consent was obtained before all study assessments. Ethics approval was obtained at the University of California, San Francisco (UCSF). This study was registered at ClinicalTrials.gov (Identifier NCT 02613273).

Participants were recruited from patient lists and physician referrals, with a focus on those living within 3 h drive of UCSF. The study protocol designed in 2016 specified onsite supervised exercise 3/week at the UCSF Mission Bay campus, but travel presented a barrier to accrual. The protocol was updated in 2017 to offer onsite supervised or remotely monitored programs. Because of the popularity of the remote option, only the remote option was offered from 2018 to 2020. The pilot study was planned to enroll 39 participants; however, investigators decided to end enrollment in March 2020 due to the COVID‐19 pandemic and the closure of gyms. Three participants were assigned to the onsite supervised program (2 aerobic, 1 resistance; 2 completed, one moved out of state after randomization) and 15 participants were assigned to the remotely monitored program (8 aerobic, 7 resistance; all 15 (100%) completed). Due to low enrollment in the supervised mode and the novelty of the remote strategy, the remote participants are the focus of this paper.

### 2.2 Randomization, stratification, and allocation concealment

Following completion of screening, consent, and baseline procedures, men were randomized 1:1:1 to aerobic exercise, resistance exercise, or usual care with block sizes of 1–2 using the R package blockrand (https://cran.r‐project.org/web/packages/blockrand/blockrand.pdf). Patients were stratified based on active therapy (abiraterone, enzalutamide, chemotherapy, etc.) at the time of enrollment (yes/no). Allocation sequences were uploaded to Research Electronic Data Capture (REDCap)^12^ and schema was concealed from the coordinator assigning participants.

### 2.3 Remote exercise program implementation

All men completed their exercise prescription at an exercise facility near their residence and visited UCSF at baseline and 12 weeks. We provided a “concierge service” to identify suitable gyms. We confirmed an employee was present most of the time and the gym was open at least 3 days per week, verified specific equipment to perform the exercises, and confirmed whether a gym waiver was signed and the amount of the monthly fees. Gym staff/trainers were not part of the study and did not work with participants; however, we verified that if the patient had a question with a particular piece of equipment, for instance, the staff would be available to help. The gym personnel activating the membership knew that the participant was part of a research study. The study paid for a 3‐month membership. We asked men to exercise at a gym to ensure that they had access to suitable equipment for the resistance training and gym staff to reduce safety concerns, given all men had mCRPC. All men were asked to exercise 3 days a week (weekends allowed) with at least a day of rest in between; participants separated sessions by 24–48 h of rest between training the same muscle groups. In addition, the participants were counseled to follow their prescription, not to do anything else new, and not to ask gym staff for additional exercises.

During the baseline visit, intervention participants received a Polar HR strap (H10) and were remotely monitored multiple times weekly by the exercise physiologist (EP) through Polar accounts using study‐generated email addresses. Participants set up the Polar Beat app on their smartphone using the study account (*N* = 14) or manually tracked Polar heart rate data using paper logs (*N* = 1). Before each prescribed exercise session, participants completed an online survey, reporting hours of sleep the night prior, resting heart rate, bone pain, fatigue, mood, motivation, and muscle soreness. They completed a post‐exercise survey assessing total adherence versus modifications to prescribed workouts (more or less sets, reps and/or weight); session RPE; session tolerance; and mood.

Participants were instructed to wear the HR monitor for all prescribed sessions. Pre‐ and post‐exercise session surveys were reviewed daily by the EP for compliance and adverse events (AEs). Weekly check‐ins were scheduled for Thursday or Friday via email or phone, with additional calls if concerns requiring more communication were observed.

### 2.4 Control arm

The control arm received usual standard of care from baseline to 12 weeks. At 12 weeks, they received their choice of an aerobic or resistance exercise program, personalized based on their 12‐week testing data, and their exercise test summary results (baseline and post‐control period test).

All arms received diet and exercise booklets, psychosocial support material, and a $50 gift card after the 12 weeks of intervention.

### 2.5 Exercise programs

The aerobic exercise intervention aimed to meet the American College of Sports Medicine (ACSM) guidelines of at least 20–30 min of moderate‐to‐vigorous intensity exercise 3–5 days/week.^13^ Exercise prescriptions were tailored to each participant's baseline cardiorespiratory and strength assessments and consultation with the treating medical physicians (e.g., reducing weight‐bearing resistance exercises if the patient had bone metastasis as a safety modification) (File S2). The aerobic training prescription, all completed on a cycle ergometer, was based on results of a baseline maximal steep ramp test.^14^ The program was designed to be vigorous and included 2 days of high‐intensity interval training (HIIT) sessions and 1 day of a moderate‐intensity continuous exercise session to balance out exertion levels and provide enough rest and recovery, given the age and limitations of the study population. The resistance training prescription was established on baseline 1‐repetition maximum (1‐RM) tests. Training sessions progressed from 1 to 4 sets of 4–15 repetitions. Exercise selection was modified in consultation with the EP throughout the program in response to participant feedback (e.g., tolerability, bone pain, etc.).

### 2.6 Data collection

Primary outcomes were intervention feasibility (attendance, adherence to the exercise prescription, and tolerance), safety (adverse events [AEs]), and acceptability (assessed via interview). Attendance equals the number of exercise sessions attended out of 36 planned sessions. Adherence equals the number of sessions completed as or more than prescribed out of 36 planned sessions. Adherence to the prescription was assessed by the EP reviewing the HR data in the Polar accounts each week. Based on meeting HR targets and session durations, the EP recorded whether they adhered or not to the prescription (yes/no) and if not, did more or less as prescribed (more time/exceed heart rate or less time/did not hit heart rate). For resistance exercise, to reduce participant burden we did not require the participants to record the exact sets x reps x load on the sessional surveys, but to indicate if they did more or less of the prescribed exercise, and if yes, then indicate whether they did more or less sets, reps, and weight. The EP discussed the surveys and Polar data with the participant on weekly calls and made a final adherence assessment. Tolerance was measured at each post‐training survey (0–10 scale: 0 = intolerable and 10 = highly tolerable).

Secondary outcomes included fatigue and bone pain measured using visual analog scales at each exercise session, number of participants reporting use of opiate pain medication, and changes in physical function and strength, which were measured at baseline and 12‐week study visits. QOL was also a secondary outcome but not the focus of this paper. Timed fitness measurements included stair climb, 400‐m walk, and repeated sit‐to‐stand.^15^, ^16^ For resistance testing and initial training, 1‐RM tests included the following exercises: chest press, leg press or extension, and seated row.

Research blood and urine and standard of care blood measurements were also collected, and participants completed surveys on lifestyle, diet, and QOL.^12^ Exploratory outcomes included the Halabi nomogram score (prognostic model for overall survival in mCRPC patients) and its components,^11^ median progression‐free survival, median overall survival, and median time to first occurrence of symptomatic skeletal‐related events.

A post‐study remote exercise feedback interview was added to the protocol in December 2019 and completed by 67% (*n* = 10) of remote intervention participants (5 aerobic; 5 resistance). The median time from the end of study to interview was 6 months (IQR 4.8, 13.8).

### 2.7 Statistical analysis

**FIGURE 1:**
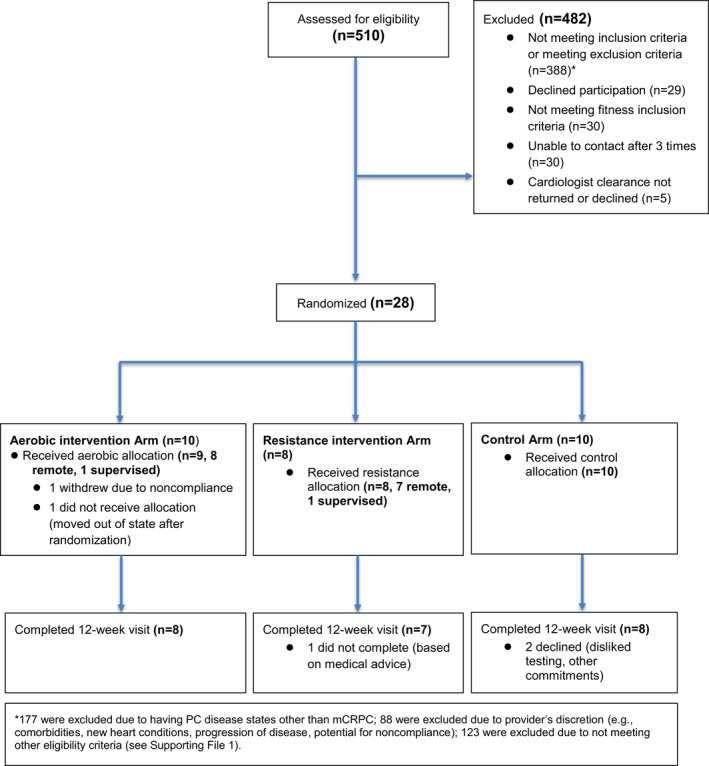
CONSORT diagram of recruitment and loss to follow‐up during the trial

The main study results (except for our enrollment summary and CONSORT Figure 1) are presented for the remote exercise intervention and control patients only (*N* = 25). Patients’ demographics and clinical characteristics were described using medians and interquartile ranges (IQR) for continuous variables and *N* with percentage for categorical variables. We used descriptive statistics to summarize results from the feedback. To assess preliminary efficacy of the interventions, absolute change from baseline to 12 weeks for the exercise tests, and Halabi score and its blood‐related components were compared between groups (aerobic vs. control, resistance vs. control and aerobic vs. resistance) using the Wilcoxon rank‐sum tests. Statistical analyses were performed using SAS version 9.4 and R version 4.0.3.

## 3 RESULTS

### 3.1 Enrollment and follow‐up

Following ethics approval at UCSF, 510 men were assessed for eligibility between 4 March, 2016 and 20 March, 2020. Recruitment and loss to follow‐up are detailed in Figure 1. Thirty‐five percent (*n* = 177) were excluded due to having PC disease states other than mCRPC, while 17% (*n* = 88) were excluded due to provider's discretion (e.g., comorbidities, new heart conditions, progression of disease, and potential for noncompliance). Five percent (*n* = 28) of those screened were consented and randomized. Eighty‐two percent of randomized participants (*n* = 23) completed the study (Figure 1). Eighty‐six percent of randomized participants (*n* = 24) completed baseline and 12‐week surveys.

### 3.2 Participant demographics for the remote intervention patient and control groups (*N* = 25)

Median age of participants was 71 years (range = 51–84). Median BMI was 28.7 kg/m^2^ (range = 22.6–36.7); 36% were obese (Table 1). Twenty percent self‐identified in a racial/ethnic minority group. Eighty‐four percent had a 4‐year university education or higher. Eighty‐four percent were married or in a civil partnership. Median distance from their residence to UCSF was 68 miles.

**TABLE 1:** Baseline characteristics of randomized remote aerobic and resistance exercise and control study participants, *n* (%) or median (IQR)

	Remote aerobic (*n* = 8)	Remote resistance (*n* = 7)	Control (*n* = 10)	Overall (*n* = 25)
Demographics				
Age, years	70 (68, 72)	73 (70, 78)	72 (66, 79)	71 (67, 75)
Race				
White	6 (75)	5 (71)	8 (80)	19 (76)
African American/Black	1 (13)	1 (14)	1 (10)	3 (12)
Asian	1 (13)	0 (0)	0 (0)	1 (4)
Other	0 (0)	1 (14)	0 (0)	1 (4)
Declined to answer	0 (0)	0 (0)	1 (10)	1 (4)
Education				
High school or less	1 (13)	1 (14)	0 (0)	2 (8)
2‐year college	2 (25)	0 (0)	0 (0)	2 (8)
4‐year college	1 (13)	2 (29)	5 (50)	8 (32)
Grad./prof. school	4 (50)	4 (57)	5 (50)	13 (52)
Employment status				
Full‐time	0 (0)	1 (14)	1 (10)	2 (8)
Part‐time	1 (13)	1 (14)	1 (10)	3 (12)
Retired	6 (75)	5 (71)	7 (70)	18 (72)
Other	1 (13)	0 (0)	1 (10)	2 (8)
Relationship status				
Married/civil partnership	8 (100)	6 (86)	8 (80)	22 (88)
Single/divorced/widowed	0 (0)	1 (14)	2 (20)	3 (12)
Distance from study site, km	76 (27, 98)	92 (73, 231)	23 (4, 45)	68 (15, 92)
Anthropometrics				
Body mass index, kg/m^2^	31 (28, 32)	30 (26, 32)	27 (26, 29)	29 (26, 32)
Waist circumference, cm	106 (99, 112)	103 (88, 106)	103 (93, 109)	104 (94, 109)
Waist‐to‐hip ratio	1.03 (0.94, 1.04)	0.98 (0.94, 1.00)	1.02 (0.96, 1.04)	1.00 (0.96, 1.04)
Clinical				
Time since diagnosis, year	9.6 (1.7, 13.5)	12.6 (1.9, 12.8)	7.7 (4.9, 18.0)	10.5 (4.2, 14.3)
Comorbidities^a^, ^b^				
Hypertension	3 (38)	1 (14)	4 (40)	8 (32)
Hypercholesteremia	3 (38)	4 (57)	3 (30)	10 (40)
Cardiovascular disease	2 (25)	2 (29)	1 (10)	5 (20)
Diabetes type II	1 (13)	0 (0)	0 (0)	1 (4)
Osteoporosis	0 (0)	1 (14)	0 (0)	1 (4)
Osteoarthritis	1 (13)	2 (29)	1 (10)	4 (16)
Stomach/intestinal disease^c^	1 (13)	3 (43)	3 (30)	7 (28)
Depression	0 (0)	3 (43)	1 (10)	4 (16)
Anemia	1 (13)	1 (14)	2 (20)	4 (16)
Other cancer	1 (13)	0 (0)	2 (20)	3 (12)
PSA level at diagnosis, ng/mL	14.2 (6.9, 120.0)	6.0 (4.4, 28.9)	11.1 (9.5, 20.9)	10.0 (5.4, 21.2)
PSA level at enrollment, ng/mL^b^	2.5 (0.7, 20.3)	3.5 (1.0, 7.9)	10.2 (1.9, 38.0)	3.9 (1.1, 16.4)
Gleason grade				
2–6	4 (50)	4 (57)	6 (60)	14 (56)
3+4	1 (13)	1 (14)	3 (30)	5 (20)
4+3	1 (13)	2 (29)	0 (0)	3 (12)
8–10	2 (25)	0 (0)	1 (10)	3 (12)
Local therapies^a^, ^d^				
Radical prostatectomy	3 (38)	5 (71)	5 (50)	13 (52)
Radiation therapy	6 (75)	6 (86)	8 (80)	20 (80)
Metastasis at enrollment^b^				
Lymph node(s)	6 (75)	4 (57)	4 (40)	14 (56)
Bone	6 (75)	5 (71)	7 (70)	18 (72)
Lung	0 (0)	0 (0)	1 (10)	1 (4)
Systemic therapies^a^, ^d^				
LHRH analog/antagonist	8 (100)	7 (100)	10 (100)	25 (100)
Abiraterone^e^	6 (75)	3 (43)	5 (50)	14 (56)
Enzalutamide^e^	2 (25)	2 (29)	3 (30)	7 (28)
Other antiandrogen	8 (100)	5 (71)	10 (100)	23 (92)
Chemotherapy	2 (25)	1 (14)	2 (20)	5 (20)
Sipuleucel‐T	3 (38)	4 (57)	5 (50)	12 (48)
Other immunotherapy	0 (0)	1 (14)	0 (0)	1 (4)
Radium‐223	1 (13)	0 (0)	1 (10)	2 (8)
Halabi nomogram score				
Low	5 (63)	5 (71)	8 (80)	18 (72)
Intermediate	3 (38)	2 (29)	1 (10)	6 (24)
High	0 (0)	0 (0)	1 (10)	1 (4)
Lifestyle characteristics				
Smoking status				
Current	0 (0)	1 (14)	1 (10)	2 (8)
Former	3 (38)	2 (29)	4 (40)	9 (36)
Never	5 (63)	4 (57)	5 (50)	14 (56)
Light exercise, min/week	163 (75, 263)	90 (0, 140)	120 (20, 180)	120 (60, 175)
Moderate exercise, min/week	54 (0, 130)	0 (0, 120)	55 (0, 150)	30 (0, 120)
Vigorous exercise, min/week	0 (0, 0)	0 (0, 0)	0 (0, 0)	0 (0, 0)
Resistance exercise, min/week	0 (0, 15)	0 (0, 0)	0 (0, 0)	0 (0, 0)

Abbreviations: IQR, interquartile range; PSA, prostate‐specific antigen.

^a^ Categories are not mutually exclusive.

^b^ Reported by patient at time of enrollment. Reported hypertension, hypercholesterolemia, cardiovascular disease, type 2 diabetes, and osteoporosis. Other comorbidities comprising ≥10% of total population were included in table.

^c^ Stomach or intestinal diseases (e.g., acid reflux, hepatitis C, gallstones, pancreatitis, irritable bowel syndrome, and ulcer disease).

^d^ From the medical record at time of enrollment.

^e^ Eighty‐six percent of those receiving abiraterone and 86% of those receiving enzalutamide were taking this drug at baseline.

Median time from diagnosis to enrollment was 10.5 years (range = 0.9–26.3). Radiation therapy (80%) was the most common localized treatment. Fifty‐six percent were prescribed abiraterone, 28% enzalutamide, 92% received other antiandrogen therapy, 48% were prescribed Sipuleucel‐T, and 20% were treated with chemotherapy before the study. Seventy‐two percent had a low Halabi nomogram score. Most men did no vigorous or resistance exercise at enrollment, and did a median of 30 min/week of moderate exercise. Eight percent were current smokers and 36% were past smokers.

### 3.3 Feasibility of remote exercise program

Remote intervention participants attempted 93% of prescribed workouts (Table 2). Pre–post‐exercise session survey completion rate was 92% for the resistance arm and 94% for the aerobic arm. Of sessions attempted, 87% were self‐reported as having been completed as prescribed or with more sets, reps, and/or weight (88% resistance remote and 86% aerobic remote). Median tolerance across all sessions was 6 (IQR = 5, 7). Subjects found the resistance exercises (median = 7) more tolerable than the aerobic arm (median = 5). Men in both arms reported no bone pain and low fatigue levels during exercise, with the aerobic arm reporting slightly more fatigue (median = 4) than the resistance arm (median = 3). Median RPE was 6 (0–10 scale, 5 = moderate, 6 = hard, 8 = very hard), combined (IQR = 6, 8) and separately for the exercise arms.

**TABLE 2:** Safety and feasibility of remote aerobic and resistance exercise among men with mCRPC

*N* (%) or median (IQR)	Aerobic (*n* = 8)	Resistance (*n* = 7)
Dropout	1	1
Attendance at ≥70% of exercise sessions^a^	7 (88)	7 (100)
Number of sessions attended (of 36 sessions)	34 (33, 35)	35 (35, 35)
Number who completed ≥70% exercise sessions as or more than prescribed^b^	5 (63)	7 (100)
Number of sessions completed as or more than prescribed (of 36 sessions)	32 (22, 34)	30 (29, 33)
Median sessional tolerance^c^	5 (4, 7)	7 (5, 8)
Number who completed ≥70% exercise sessions with tolerance ≥5^b^	6 (75)	6 (86)
Perceived exercise intensity (session RPE)^d^	6 (5, 8)	6 (5, 7)
Perceived bone pain level^e^	0 (0, 1)	0 (0, 1)
Perceived fatigue level^f^	4 (2, 5)	3 (2, 5)

Abbreviations: IQR, interquartile range; RPE, rated perceived exertion.

^a^ Calculated out of 36 sessions.

^b^ Calculated out of sessions completed.

^c^ Scale 0–10: 0 = not tolerable, 5 = moderately tolerable, 10 = very tolerable.

^d^ Scale 0–10: 0 = no exertion, 5 = moderate exertion, 10 = high exertion.

^e^ Scale 0–10: 0 = no bone pain, 5 = moderate bone pain, 10 = high bone pain.

^f^ Scale 0–10: 0 = no fatigue, 5 = moderate fatigue, 10 = high fatigue.

### 3.4 Adverse events

No safety concerns related to the exercise programs were identified. Eight of 14 AEs were reported by men in the resistance arm (Table 3) and were most commonly joint or bone pain consistent with disease status. Three joint/bone pain AEs were classified as possibly related to the study. One man in the aerobic arm experienced hip and lower back pain, while two men in the resistance arm reported pain, one in the heel and the other in the shoulder where he had received radiation therapy >1 year prior to enrollment. The first and third patients received pain medication, and the second patient's pain resolved after a physician visit.

**TABLE 3:** Adverse events among men with mCRPC participating in a 12‐week trial of remote aerobic or resistance exercise or control

	Aerobic (*n* = 8)	Resistance (*n* = 7)	Control (*n* = 10)	Overall (*n* = 25)
Any AE	4	8	2	14
Study‐related AE	1	2	0	3
Specific AEs				
Joint or bone pain, any	1	6	1	8
Joint or bone pain, study‐related	1	2	0	3
Muscle pain/injury, any^a^	2	1	0	3
Dizziness or vertigo, any^a^	0	1	0	1
Cardiovascular event, any^a^	0	0	1	1
Other (cataract issue), any^a^	1	0	0	1

^a^ No AE’s in this category were study‐related.

### 3.5 Intervention interview results and study acceptability

Patients reported high overall satisfaction with the program, 90% being satisfied or very satisfied, 90% rating the program as very good or excellent, and 90% reporting they would recommend the study to others. Notably, 90% of participants reporting they would not have participated if the program was only available onsite (these participants completed the study prior to the COVID‐19 pandemic). Average one‐way transportation time was 10 min to their local study gym and 158 min to UCSF.

Using the HR monitors did not pose significant issues, and no one reported difficulty with completing the online surveys. However, technology literacy in the enrolled study population was high. All participants owned a computer, 70% had owned a tablet for 10 years, and 100% had owned a smartphone for an average of 13 years (Table 4).

**TABLE 4:** Acceptability of remote program

	Aerobic (*n* = 5)	Resistance (*n* = 5)	Overall (*n* = 10)
Characteristic, median (IQR), or *n* (%)			
Setup			
Usefulness of orientation^a^	4 (4, 5)	5 (4, 5)	5 (4, 5)
Problems setting up exercise facility/gym	0 (0%)	2 (40%)	2 (20%)
Challenges with setup of Polar heart rate monitor to smartphone	1 (20%)	1 (20%)	2 (20%)
Components, usefulness, and difficulty			
Usefulness of exercise record sheet^a^	4 (4, 4)	4 (4, 5)	4 (4, 5)
Difficulty recording exercise on record sheet	1 (20%)	0 (0%)	1 (10%)
Usefulness of resistance exercise picture guide^a^, ^b^	N/A	5 (4, 5)	N/A
Usefulness of Polar heart rate monitor	5 (4, 5)	5 (4, 5)	4 (3, 5)
Always or almost always wore heart rate monitor during your exercise program	5 (100%)	5 (100%)	10 (100%)
Difficulty using heart rate monitor	0 (0%)	2 (40%)	2 (20%)
Comfort of chest strap^c^	4 (3, 4)	3 (3, 4)	4 (3, 4)
Convenience of chest strap^d^	4 (4, 4)	4 (4, 4)	4 (4, 4)
Difficulty completing pre‐ and post‐exercise session surveys	0 (0%)	0 (0%)	0 (0%)
Usefulness of weekly calls with exercise specialist^a^	4 (4, 4)	5 (5, 5)	5 (4, 5)
Frequency of weekly calls was:			
Too little	0 (0%)	0 (%)	0 (0%)
Just right	4 (80%)	5 (100%)	9 (90%)
Too much	1 (20%)	0 (0%)	1 (10%)
Duration of weekly call (min)	6 (5, 15)	5 (3, 15)	6 (5, 15)
The length of the call was:^e^			
Just right	5 (100%)	5 (100%)	10 (100%)
Felt exercise specialist answered all questions	4 (80%)	5 (100%)	9 (90%)
Called exercise specialist outside of scheduled weekly call	1 (20%)	2 (40%)	3 (30%)
Usefulness of the local gym^a^	4 (4, 4)	4 (3, 4)	4 (3, 4)
Obstacles attending local gym	0 (0%)	1 (20%)	1 (10%)
Preferred workout time, AM	3 (60%)	2 (40%)	5 (50%)
Preferred workout time, PM	2 (40%)	3 (60%)	5 (50%)
Ease of attending remote sessions 3x/week^f^	4 (3, 4)	4 (4, 4)	4 (3, 4)
Ease of exercise program^f^	2 (2, 2)	3 (2, 3)	2 (2, 3)
How easy was it to			
Complete exercises prepared by exercise specialist^g^	2 (2, 2)	3 (2, 3)	2 (2, 3)
Choose other exercises to do beyond those assigned^b^, ^g^	N/A	4 (4, 4)	N/A
Remember how to do the exercises correctly^g^	4 (2, 4)	4 (3, 4)	4 (3, 4)
Remember to exercise^g^	5 (5, 5)	5 (4, 5)	5 (5, 5)
Find time to exercise^g^	4 (3, 5)	5 (4, 5)	5 (3, 5)
Find an appropriate place to do the prescribed exercise(s)^g^	5 (4, 5)	5 (4, 5)	5 (4, 5)
Stay motivated^g^	5 (4, 5)	5 (3, 5)	5 (3, 5)
Remember why exercise matters^g^	5 (5, 5)	5 (5, 5)	5 (5, 5)
Remember to use the heart rate monitor when you exercised^g^	5 (5, 5)	5 (5, 5)	5 (5, 5)
Would be able to attend exercise sessions onsite three times a week at UCSF (if no opportunity for remote program)	1 (20%)	0 (0%)	1 (10%)
Overall program rating			
Excellent	3 (60%)	2 (40%)	5 (50%)
Very good	1 (20%)	3 (60%)	4 (40%)
Good	0 (0%)	0 (0%)	0 (0%)
Fair	1 (20%)	0 (0%)	1 (10%)
Poor	0 (0%)	0 (0%)	0 (0%)
Program satisfaction			
Very satisfied	3 (60%)	4 (80%)	7 (70%)
Satisfied	1 (20%)	1 (20%)	2 (20%)
Neutral	0 (0%)	0 (0%)	0 (0%)
Dissatisfied	1 (20%)	0 (0%)	1 (10%)
Very dissatisfied	0 (0%)	0 (0%)	0 (0%)
Would recommend study to others	4 (80%)	5 (100%)	9 (90%)

^a^ Scale 1–5: 1 = not at all useful, 5 = very useful.

^b^ Resistance arm only.

^c^ Scale 1–5: 1 = very uncomfortable, 5 = very comfortable.

^d^ Scale 1–5: 1 = very inconvenient, 5 = very convenient.

^e^ None answered too short or too long.

^f^ Scale 1–5: 1 = very challenging/very hard, 5 = very easy.

^g^ Scale 1–5: 1 = very difficult, 5 = very easy.

Most participants found the short, weekly calls with exercise specialists helpful. Participants remembered to exercise, found time to exercise, found an appropriate place to do the prescribed exercise(s), and remembered to use the HR monitor when exercising, while completing the exercises was considered more difficult. Participant feedback and suggestions for next steps are summarized in File S3.

### 3.6 Secondary outcomes

#### 3.6.1 Exercise testing results

Both the aerobic and resistance arms showed mode‐specific adaptations (File S4).

The resistance arm improved more in the 1‐RM tests than the other two arms, while the aerobic arm had greater changes in the steep ramp test performed on the bike and the 400‐m walk test than the other two arms. Resting heart rate was modestly reduced in the resistance and aerobic arms and modestly increased in the control arm. For additional testing parameters, see File S4.

#### 3.6.2 Halabi score outcomes

There were no meaningful changes in absolute Halabi scores, within or between arms; and no differences in predicted 24, 36, 48‐month survival probabilities across arms (data not shown). Changes in the Halabi score components are summarized in File S5, showing 30%, 29%, and 0% of participants increasing from a lower to higher Halabi score level (poorer prognosis) for the control, resistance, and aerobic exercise arms, respectively, primarily driven by diagnosis of a new metastasis, change in ECOG status, and change in lactate dehydrogenase.

## 4 DISCUSSION

In this pilot RCT, we found that remote exercise was feasible, safe, and acceptable for men with mCRPC. The primary strength of the remote program was convenience, and the remote exercise program attendance rate was high, with a high satisfaction rate. In addition, the RPE of the exercise sessions showed that the participants were being adequately challenged (median = 6). Reported bone pain was none to low, with a median of 0 out of 10 overall, demonstrating that the program was successful in creating a tailored program that avoided patients’ metastatic sites to prevent injury. Three moderate AEs were possibly related to the study, but were more likely related to natural disease progression.

The 12‐week exercise programs resulted in expected improvements. Aerobic training improved cardiorespiratory performance and resistance training was superior for improving strength and function. As anticipated, involvement in a structured program led to routine exercise and over 90% of the workouts were attempted.

We acknowledge that many men screened out due to the provider's discretion (including having comorbidities or disease progression) or eligibility criteria. Since starting the study, a global phase III study called INTERVAL‐GAP4 (INTense exeRcise for surviVAL among men with Metastatic Prostate Cancer) was launched to study the effect of 48 weeks of supervised aerobic and resistance exercise followed by 48 weeks of self‐managed exercise on overall and progression‐free survival among 866 men with mCRPC or metastatic hormone‐sensitive prostate cancer (mHSPC) (NCT02730338).^17^ Both CHAMP and INTERVAL‐GAP4 have lengthy eligibility criteria; however, the latter expanded eligibility to include those with disease progression. Participants with angina and hypertension are eligible with physician clearance, and efforts are being made to support exemption requests if the patient is well‐suited but fails to meet the select criteria. This is important to increase generalizability of the findings.

Remotely monitored behavioral interventions are gaining importance, especially in geographic areas where travel‐related barriers such as high traffic congestion negatively impact participation, and we obtained meaningful feedback from participants to improve adherence to these interventions. CHAMP required use of a Polar HR monitor, the Polar app (or paper log), and web‐based surveys, and the participants’ high technological literacy may have contributed to the success of the program. Future programs must consider the literacy level of the target population to adapt study onboarding as needed. Some participants still had intermittent trouble with the technology, so additional support and resources must also be available. Further tailoring of the weekly call with the exercise specialist and bringing patients onsite during the program for personalized training and evaluation may be useful. Although there could have been differences with the personnel employed at each local gym, the study was not designed around gym staff and relied solely on the study's exercise physiologist for the exercise prescription and coaching, which limited the gym‐related requirements and helped to ensure standardized feedback for all intervention participants.

There were many competing priorities with the intervention, including personal travel, life events, symptom progression, and cancer recurrence, which affected participants’ perception of difficulty of the exercise program. Participants were happy with their local gym experience with remote monitoring, which gave them flexibility and required minimal travel. In future studies, the use of a timer app for work rest ratios and provision of additional feedback on progress during and at the end of the program may increase participants’ success and study satisfaction. The study eliminated cost barriers to exercise during the study period, but did not solve the long‐term need for access to facilities. Future iterations of these interventions could involve long‐term collaborations with gyms to provide free or low‐cost gym memberships to cancer patients and/or survivors, such as the Young Men's Christian Association (YMCA or Y) programs. Providing a guide and home exercise equipment could be feasible based on patient feedback, though further study is required to assess the safety and feasibility of remote interventions for metastatic patients in the home setting. Virtual, home‐based interventions would allow the exercise specialist to supervise participants while completing their exercise prescription and remove the need for a public gym; while a remote monitoring format utilizing local gyms may improve access to equipment that may not be feasible to buy for virtual, home‐based interventions.

Two studies, both 12 weeks, intentionally enrolled metastatic PC patients with bone metastases to supervised resistance or multimodal exercise, and reported that exercise was safe and well‐tolerated.^9^, ^10^ Our study focused on remote self‐monitored aerobic or resistance exercise and reported that exercise is safe and well‐tolerated when performed independently, with the weekly guidance of an exercise specialist. More recent studies successfully incorporated hybrid formats. Bourke et al. conducted a 3‐month randomized trial of a combined aerobic and resistance + diet program versus control in 100 men on ADT for locally advanced (*N* = 80) or metastatic (*N* = 20) PC.^18^ The program was tapered with two supervised sessions and one self‐directed session in weeks 1–6 and one supervised session and two self‐directed sessions in weeks 7–12. Adherence was 94% for the supervised and 82% for the independent exercise sessions during the 12 weeks, with durability observed at 6 months in fatigue and exercise behavior.^18^ Utilizing hybrid or tapered approaches may be more feasible, and may help to increase self‐efficacy and long‐term exercise habits, after the study ends.

A few studies, like CHAMP, are also integrating activity trackers, for participant use and feedback.^19^, ^20^, ^21^ Cadmus‐Bertram et al. enrolled 50 non‐metastatic breast and colorectal cancer survivors to receive a survivorship care plan +/− a 12‐week multicomponent physical activity module with the goal to increase moderate‐to‐vigorous physical activity (MVPA) to 150 min/week and daily steps to 10,000.^19^ The module included a Fitbit (that integrated physical activity data into the electronic health record (EHR) for clinician review) and customized email feedback from a coach (4 times in 12 weeks). The study reported improved physical and mental health, sleep, exercise self‐efficacy, MVPA (69 min/week vs. 20 min/week), and steps (average increase of +1470 vs. −398 steps) in intervention versus control.^19^, ^22^ Exercise integrations with the EHR or a participant/coach portal, or the use of a physical activity app + website provide more opportunity, flexibility, and support for remote‐based exercise interventions. Furthermore, incorporating exercise specialists in remote‐based studies of advanced cancer patients with additional comorbidities is warranted, would promote safe exercise, and may be less costly to implement than fully supervised programs incorporating exercise physiologists or trainers. To our knowledge, no other study in cancer survivors has used exercise specialists in a remote capacity to regularly monitor remote exercise based on individual exercise prescriptions except for the Active Surveillance Exercise Clinical Trial (ASX) study, which is ongoing in localized PC patients (NCT02435472).

These CHAMP results guided the addition of the CHAMP remote exercise protocol to the INTERVAL‐GAP4 trial protocol and affiliated sites are poised to launch this format at the end of 2021. With the addition of the CHAMP remote intervention option, INTERVAL participants can now choose remotely monitored exercise as an alternative option to onsite supervised exercise. The study is open at 18 sites (with an additional 4 pending) in 8 countries, and we expect that the addition of the CHAMP remote exercise protocol, which will be fully implemented as gyms reopen when safe (closed due to COVID‐19), will allow additional sites to join and increase enrollment rates, especially in locations where travel time is a barrier.

There are several limitations to consider. The study was terminated early, and therefore the sample size was smaller than planned. Although we observed improvements in fitness over the intervention period, we recognize that baseline values were dissimilar across groups, which occurred by chance due to the small sample size. The study was designed to examine the primary outcomes of feasibility, safety, and acceptability and was not powered to assess the effects of the fitness‐related secondary outcomes. The intervention was limited to 12 weeks in duration, and longer term studies like INTERVAL are needed to evaluate whether the high adherence we observed can be maintained over time. Providing long‐term support as patients progress may also have beneficial effects on functional outcomes and QOL. Two thirds of patients in the remote exercise program were stable, while the remainder were progressing clinically. Lastly, most patients were White, married, and highly educated. Future studies evaluating the feasibility of remote exercise training in populations with more racial/ethnic and socioeconomic diversity are warranted.

In this pilot RCT, exercise completed with remote supervision by an EP was feasible, safe, and acceptable for men with mCRPC. Based on our findings we provide direction and rationale for future studies to determine the effect of exercise on treatment toxicity, cancer symptoms, QOL, and clinical outcomes for people living with metastatic cancer.

## CONFLICT OF INTEREST

None related to the research.

## ETHICAL APPROVAL STATEMENT

Ethics approval was obtained at the University of California, San Francisco (UCSF).

## Supporting information

File S1–S5

## Data Availability

The data that support the findings of this study are available from the corresponding author upon reasonable request.
