# Supplementary material for: Feasibility, safety, and acceptability of a remotely monitored exercise pilot CHAMP: A Clinical trial of High‐intensity Aerobic and resistance exercise for Metastatic castrate‐resistant Prostate cancer
Source: Cancer Med. 2021 Oct 12;10(22):8058–70. doi: 10.1002/cam4.4324 (PMC8607248; doi:10.1002/cam4.4324)
Supplement: Supplementary file 1 — File S1–S5 [file CAM4-10-8058-s001.docx]

**Supporting file 1.** CHAMP Eligibility Criteria

### Inclusion Criteria

- Histologically documented adenocarcinoma of the prostate with systemic bone or node metastatic disease despite castrate levels of testosterone (<50 ng/dL) due to orchiectomy or LHRH agonist. Castrate levels of testosterone must be maintained while on study. Men can be enrolled prior to starting abiraterone and/or enzalutamide OR already be receiving treatment with abiraterone and/or enzalutamide.
- Be on androgen deprivation therapy (ADT) with a GnRH agonist/antagonist or prior bilateral orchiectomy.

All patients will be required to be on ADT during the study period.

- ≥4 weeks since last major surgery and fully recovered.
- Age ≥18 years
- ECOG performance status 0-1
- Be able to complete a steep ramp test
- Physician consent to participate in vigorous aerobic or resistance exercise training.
- Required Initial Laboratory Values:
  - - Platelet count ≥ 100,000/uL
    - Hepatic and renal dysfunction that would preclude participation in an exercise program, in the opinion of the treating physician
    - Serum testosterone ≤ 50 ng/dL

### Exclusion Criteria

- Men with small cell neuroendocrine tumors or features of small cell disease
- Any prior chemotherapy for castrate-resistant prostate cancer

* Metastatic CRPC pre-chemo (metastatic castrate-resistant adeno prostate cancer, pre-CRPC chemotherapy), which is given for hormone-sensitive prostate cancer, is allowed. Metastatic CRPC post- chemo (metastatic castrate-resistant adeno prostate cancer, post-CRPC chemotherapy) is allowed with the treating physician’s (oncologist) discretion/approval.

- History of hypertension that is not well-controlled (≥160/90) on anti-hypertensive therapy
- Any contraindications to vigorous exercise, including, but not limited to: brain metastases; current congestive heart failure (New York Heart Association Class II, III or IV); serious or non-healing wound, ulcer, or bone fracture; spinal cord compromise or instrumentation due to metastatic disease; peripheral neuropathy ≥grade 3. No serious cardiovascular events within 12 months including, but not limited to, transient ischemic attack (TIA), cerebrovascular accident (CVA), or myocardial infarction (MI).
- Experiences shortness of breath, chest discomfort, or palpitations when performing activities of daily living (patient with these symptoms can participate in the study with cardiologist clearance)
- Has difficulty climbing a flight of stairs due to physical impairment
- Has been told by a doctor to have a heart condition and recommended only medically supervised activity
- Has chest pain brought on by physical activity (patient can participate in the study with cardiologist clearance)
- Has developed chest pain in the past month (patient can participate in the study with cardiologist clearance)
- Any serious or non-healing wound, ulcer, or bone fracture.
- Any spinal cord compromise or instrumentation due to metastatic disease. Radiation therapy for metastatic disease is allowed.
- Any peripheral neuropathy ≥grade 3.
- Moderate-to-severe bone pain (i.e., National Cancer Institute’s Common Terminology Criteria for Adverse Events grade 2-3 bone pain).
- Men participating in vigorous exercise for 75 minutes or more per week, and/or structured resistance exercise on three or more days per week are not eligible.
- Men who do not complete the baseline lifestyle and quality-of-life questionnaires and FFQ will not be eligible.

**Supporting file 2.** Summary of Aerobic and Resistance Exercise Programs

Aerobic Exercise Programs (actual ranges for exercise specified below):

- **Day 1**
  - **Assessment:** Fatigue, bone pain visual analog scale (VAS), resting heart rate
  - **Warm up:** 5 minutes cycling at a light to moderate intensity
  - **Exercise:** 5 to 16 minutes of high-intensity interval training (participant prescribed RPE for work interval equal to 5, 6, 7, 8, or 9 on the RPE scale (0-10), aiming to achieve 85% of maximum heart rate (HRmax))
  - **Cool down:** 5 minutes cycling at a light to moderate intensity
  - Trunk flexion and extension*, stretching
  - **Assessment**: Adherence to prescription, level of exertion, tolerance

## Day 2

- - **Assessment:** Fatigue, bone pain VAS, resting heart rate
  - **Warm up:** 5 minutes cycling at a light to moderate intensity
  - **Exercise:**  10 to 50 minutes of continuous moderate and vigorous intensity aerobic exercise training (45-81% HRmax)
  - **Cool down:** 5 minutes cycling at a light to moderate intensity
  - Trunk flexion and extension*, stretching
  - **Assessment**: Adherence to prescription, level of exertion, tolerance

## Day 3

- - **Assessment:** Fatigue, bone pain VAS, resting heart rate
  - **Warm up:** 5 minutes cycling at a light to moderate intensity
  - **Exercise:**  6 to 33 minutes high-intensity interval training (participant prescribed RPE for work interval equal to 5, 6, 7, 8, or 9 on the RPE scale (0-10), aiming to achieve 85% of maximum heart rate (HRmax))
  - **Cool down:** 5 minutes cycling at a light to moderate intensity
  - Trunk flexion and extension*, stretching
  - **Assessment**: Adherence to prescription, level of exertion, tolerance

*modified in patients with lumbar or pelvic lesions

Resistance Exercise Programs: prescription prior to modifications for individual participants

## Day 1

- - **Assessment:** Fatigue, bone pain VAS, resting heart rate
  - **Warm up:** 5 minutes cycling at a light to moderate intensity
  - **Exercise:** High load, low volume resistance training – build from 1 to 2 sets over first two weeks to 3 sets at a load of 4-10 RM (rests 90-120 sec)
  - **Cool down:** 5 minutes cycling at a light to moderate intensity

## Trunk flexion and extension*, stretching

- - **Assessment**: Adherence to prescription, level of exertion, tolerance

## Day 2

- - **Assessment:** Fatigue, bone pain VAS, resting heart rate
  - **Warm up:** 5 minutes walking at a light to moderate intensity
  - **Exercise:** Light load, high volume resistance training – build from 1 to 4 sets at a load of 10-15 RM (rests 30-60 sec)
  - **Cool down:** 5 minutes cycling at a light to moderate intensity
  - Trunk flexion and extension*, stretching
  - **Assessment**: Adherence to prescription, level of exertion, tolerance

## Day 3

- - **Assessment:** Fatigue, bone pain VAS, resting heart rate
  - **Warm up:** 5 minutes cycling at a light to moderate intensity
  - **Exercise:** Moderate load, moderate volume resistance training – build from 1 to 4 sets at a load of 8-12 RM (rests 30-90 sec)
  - **Cool down:** 5 minutes cycling at a light to moderate intensity
  - Trunk flexion and extension*, stretching
  - **Assessment**: Adherence to prescription, level of exertion, tolerance

*modified in patients with lumbar or pelvic lesions

*Additional exercise program information*

Training was periodized within cycles of both 7 days (microcycle) and 28 days (mesocycle). To achieve periodization across the mesocycle, intensity of both aerobic and resistance training increased with matching decreases in volume. On the first training day of each mesocycle, the intensity was dropped, volume increased, and the pattern repeated across the mesocycle. For example, within each mesocycle, training weight progressed linearly during the first 14 days, exercise volume was dropped during days 15-21, and the cycle finished with an unloaded period from days 22-28.

Autoregulation of training was applied to enhance adherence and tolerance to the prescribed protocol and achieve the target exercise mode, volume, and intensity across each cycle. Using this method, the participant, in conjunction with their EP, could adjust intensity and volume of the session according to their perceived capacity at that time. For example, on a day when the participant felt highly fatigued, they could choose to complete their exercises at a lower intensity. Remote monitoring of autoregulation entailed a review of exercise volume completed based on pre- and post-exercise surveys.

We attempted to reschedule missed sessions within the same week, while allowing at least 48 hours between exercise sessions of the same muscle group. In any one week, three exercise sessions were allowed. The number of and reasons for missed sessions were recorded. Up to 3 sessions could be made up after the 12-week period. If a participant missed ≥3 consecutive sessions due to a medical reason, up to 12 sessions could be made up, within an additional 28 days, if the patient agreed.

*Aerobic exercise intervention program*

The aerobic exercise prescription, comprised of both interval training and continuous sessions, was prescribed based on the results of the Steep Ramp test completed at the baseline visit and levels of exertion. RPE was used for the intensities prescribed for the interval training sessions ranging from an exertion of 5 to 9. Maximum heart rate from the Steep Ramp was used to determine the intensities of the continuous exercise sessions (heart rate zones) used in the program ranging from 45% to 81% of HRmax. To ensure a safe introduction into HIIT, the initial exercise sessions were completed at low volume and moderate intensity and slowly built up as the weeks progressed. For Weeks 1-4, Day 1 (short work intervals) exercise prescribed progressed from 9 minutes to 14 minutes with an RPE increasing from 6 to 8; work interval: 20-30 seconds/rest interval: 90-120 seconds. Day 2 (continuous exercise) exercise progressed from 15 minutes to 25 minutes at 45% to 80% of HRmax. Day 3 (long work intervals) exercise prescribed progressed from 14 minutes to 28 minutes with an RPE increasing from 5 to 8; work interval: 120-240 seconds/rest interval: 60-90 seconds. For Weeks 5-8, Day 1 exercise prescribed progressed from 15 minutes to 16 minutes with an RPE of 8; work intervals: 30-60 seconds/rest intervals: 90 seconds. Day 2 exercise prescribed progressed from 30 minutes to 40 minutes at 66% to 80% of HRmax. Day 3 exercise prescribed progressed from 28 minutes to 33 minutes with an RPE of 8; work intervals: 240 seconds/rest intervals: 90 seconds. For Weeks 9-12, Day 1 exercise prescribed progressed from 15 minutes to 16 minutes with an RPE increasing from 8 to 9; work intervals: 30-60 seconds/rest intervals: 90 seconds. Day 2 exercise prescribed progressed from 40 minutes to 50 minutes at 61% to 81% of HRmax. Day 3 exercise prescribed progressed from 28 minutes to 33 minutes with an RPE increasing from 8 to 9; work intervals: 240 seconds/rest intervals: 90 seconds.

*Resistance exercise intervention program*

Resistance training exercises were individually prescribed based on baseline 1RM chest press, leg press, leg extension and seated row assessments. This training consisted of a prescribed load for each session building from 1 to 4 sets and repetitions of each exercise ranging from 4 to 14. A total of eight exercises were performed each day - five exercises were pre-selected for the participants, and they were required to select three more from the exercise booklet provided and received approval from the exercise physiologist prior to beginning. All major muscle groups were targeted in the program, with the exception of areas the doctor had reported to avoid, which included chest, legs, back, arms and core. Exercise machines and free weights were both used in the program. During cycle 1, resistance exercise volume was introduced incrementally, starting with 1 x 8RM. During subsequent cycles, the resistance training prescription followed a standard linear progression. Exercises were modified by the EP and the participant’s physician to avoid areas of metastasis or pain. If the participant was to determine the load on the additional exercises they were told to perform and were prescribed 8RM for that session, it was instructed that the self-selected weight should only be lifted for a maximum of 8 repetitions with safe and proper form. For Weeks 1-4, sets increased from 1 to 3 and reps ranged from 8 to 15. For Weeks 5-8, 3 sets were performed with reps ranging from 6 to 15. For Weeks 9-12, sets increased from 3 to 4 and reps ranged from 4 to 15.

**Supporting File 3:** Remote program feedback and next steps

|  | Aerobic | Resistance | Potential next steps: |
| --- | --- | --- | --- |
|  |  |  |  |
| **Technology interaction** | "Not super expert but not incompetent"  “40-year career in semiconductor business”  “I can go online and send emails” | “Getting better, B+”  “Former VP of engineering, so daily interactions [with technology] are pretty standard” | Continue to provide onboarding experience before app-based intervention |
| **Challenges setting up** |  | “Had some intermittent trouble setting up with HR monitor--unable to pair a couple days so couldn't record those days. May have been my smartphone at the time, an older LG…Had to be in different health clubs, a couple times I was at UCSF because of treatments. sometimes a hotel.” | Provide an on-demand support resource |
| **Polar Heart Rate Monitor** | “Wished I could keep it after the study--was irritated about that”  **“**Kind of cool…automatic readback”  “Liked it—I want to get one” | “Only unable to sync three times…my phone fell on some metal out of my loose pocket--it cracked, and I had to get it replaced before the next exercise day”  “Liked it enough to buy one myself”  “Great until week 4-- the one that I got kept running out of batteries. Replaced a few batteries until [the monitor] died, had to buy my own” | Ensure functional heart rate monitor  Budget for extra resources (e.g., batteries) |
| **Weekly exercise session surveys** | “Helpful in keeping me motivated”  “Exercise record sheet is a little confusing: what are the checkmarks for? and also the seconds versus minutes was confusing…I'd like to do another one…This really became part of my routine” | “Good” |  |
| **Weekly call with exercise specialist** | “[She] was encouraging and helpful and easy to work with”  “It was more small talk; it wasn't life or death” | “Very helpful”  “Fine; he was a really nice guy” |  |
| **Difficulty of exercise program** | “Coupled with moving and putting the house on market, there were some weeks where I only got 2 [sessions in] but I would sneak in an extra day on weekends to make up for it-- normally it would have been easy” | “I was traveling a lot for treatments”  “If I had not had recurrence, I could have finished the study” |  |
| **Gym experience** | “Good; I was familiar with the gym when I went into the study; after clarifying with [the exercise specialist], I found the right machine, and things were smooth from there”  “Good--we'd been there before” | “Had to travel around a lot for treatments and ended up working up in a couple of different gyms. My main gym was servicing one of the machines I used so I had to travel a little farther for a gym”  “Good—I was able to set up one gym in each place I live.  “Top-of-the-line gym 5 minutes away: Bay Club. I had a great set-up. Sunflower was previously a low-cost program for cancer patients created; no longer a part of the Bay Club which now has a special program for cancer patients, where they go for free.” | Collaboration with local gyms providing discounts or free gym memberships for cancer survivors |
| **Timing aerobic exercises** | “Having the time recommendations for exercises in minutes instead of seconds because I used a watch to time myself--that was the hardest part for me”  “If you could create an app [to time the aerobic exercises] that would be great--say I has to do 6 cycles of 2 min intense, rest for 90 sec—I lose count” |  | Recommend a timer app to complement exercise recommenda-tions |
| Post-intervention continuity | “Lost 25 pounds during the study--felt really good, I need the motivation-- being on ‘female hormones’ for 14 years, I have no muscle left. I would stay up on the cardio if doing resistance, since I’m already at the gym and already committed to the study. It gives the cancer patient the full spectrum and knowledge of cardio and resistance--I had wanted the resistance and take it to the next level.”  “I may have preferred more on-site sessions at maybe 3 weeks, 6 weeks, etc. to work with the exercise physiologist for personalized training/evaluation.  “Did not get the personal training I hoped for”  “I feel like I gave more than I got…thought I would get an evaluation at the end of the program for how I did and what that might mean but did not get that…had believed there would be more continual feedback…”  “I’m wondering the trend lines of my blood work”  “After the end of the study, Nicole gave me the regimen for the other branch of the study, and I wasn't as committed to doing that. I went to the wrong gym for that--so I didn't follow through even though I wanted to--if the study had gone on longer, for another month or two at the more expensive gym” | “I never got any feedback for how my group did--hadn't heard anything since participating…in the last few months, because of treatment, I wouldn’t be able to do the study because I’m using a walker now. My legs are very weak now due to severe lymphedema. Exercise was an important part of the total treatment, and it's very important. I’m trying to think of ways to exercise nowadays. Would really like to hear how the study went when published.”  “Would like feedback on how the study goes” | Disseminate manuscript to study participants once published  Design exercises that are accessible for those with progressive prostate cancer symptoms i.e., lymphedema |
| Disappoint-ment | “Did not meet my personal expectations/goals, even in the context of knowing that I was not capable of some of the exercises provided… biggest disappointment was not getting a program that would give me a document/orientation to the resistance program to engage in…I may have been unrealistic…learned I wasn't as capable as I thought I was. I hope that I benefitted others and would recommend it from that standpoint.” |  | Improve tailoring to participant’s individual goals and capabilities |
| “What did you like about CHAMP?” | “It was convenient, the gym was free, and I lost 30 pounds”  “Motivational, keep track of progress over time”  “Didn't have to travel”  “Convenient”  “It was nice to go to the post- meeting with [the exercise specialist], seeing that everything improved” | “It was convenient. I was glad to be randomized to the strength training group since I’ve been doing aerobic exercise my whole life and was biking 200 miles a week before prostate cancer diagnosis. This was the first time I'd done some different exercise stuff”  “Didn't have to go to hospital, could go to own gym which made it handy”  “It was convenient, doing it at my own gym was easy, controlled my own time”  “I did very well, I increased my reps, and increased how much I could bike…I liked the pre- and post-tests, I’m competitive so this was in my wheelhouse at the time--I'm competing with a bunch of old guys with cancer!”  “It was fine, it fit perfectly with who I was and made me go to the gym--I even exercised in Maui"  “A program like this should be recommended to others with metastatic prostate cancer”  “I think it was pretty close to perfect" |  |

| **Supporting File 4.** Estimated effect of remote aerobic or resistance exercise on physical performance in men with mCRPC* | | | | | | | | | |
| --- | --- | --- | --- | --- | --- | --- | --- | --- | --- |
|  | Control | | Resistance | | | Aerobic | | | |
|  | Baseline Median (IQR) | Median Change (IQR)^a^ | Baseline Median (IQR) | Median Change (IQR)^*^ | Median Difference from Control  (95% CI) | Baseline Median (IQR) | Median Change (IQR)^*^ | Median Difference from Control (95% CI) | Median Difference from Resistance (95% CI) |
| Resting HR | 73.5 (67.8, 79.2) | 1.0 (-1.2, 7.5) | 70.0 (66.0, 78.0) | -1.5 (-6.2, 3.2) | -2.5  (-12.0, 9.0) | 67.0 (59.8, 77.8) | 0.0 (-3.5, 3.0) | -1.0  (-10.0, 8.0) | 1.5  (-8.0, 10.0) |
| Stair Climb Test: Time, seconds | 8.6 (7.0, 9.2) | -0.7 (-1.2, 0.0) | 7.2 (6.4, 11.5) | -0.1 (-0.2, -0.1) | 0.5  (-0.4, 2.0) | 7.4 (6.5, 9.9) | -0.2 (-0.8, 0.4) | 0.5  (-1.0, 1.8) | 0.0  (-1.9, 0.8) |
| 400m Walk Test: Time, seconds | 280.0 (252.2, 317.2) | -6.0 (-14.0, -3.0) | 271.0 (251.5, 332.0) | -5.0 (-18.0, 3.5) | 1.0  -14.0, 24.0) | 287.0 (246.2, 326.5) | -11.0 (-15.0, 13.5) | -5.0  (-14.0, 47.0) | -6.0  (-24.0, 39.0) |
| 400m Walk Test: RPE | 6.0 (5.2, 6.8) | 0.5 (-1.5, 1.2) | 5.0 (5.0, 7.5) | 1.0 (1.0, 3.2) | 0.5  (-1.0, 5.0) | 5.5 (5.0, 6.0) | 1.0 (0.5, 2.0) | 0.5  (-1.0, 4.0) | 0.0  (-3.0, 2.0) |
| 400m Walk Test: Max HR, bpm | 145.0 (125.0, 152.0) | 8.0 (-3.0, 23.5) | 133.0 (118.0, 157.0) | 5.0 (1.2, 8.8) | -3.0  (-24.0, 12.0) | 127.0 (106.0, 135.0) | 0.0 (-9.5, 7.0) | -8.0  (-40.0, 7.0) | -5.0  (-29.0, 8.0) |
| 400m Walk Test: 1-Minute HR | 119.0 (108.0, 125.2) | -2.5 (-6.0, 7.8) | 121.0 (97.5, 134.0) | 5.0 (-0.5, 6.0) | 7.5  (-9.0, 18.0) | 104.5 (94.8, 111.0) | 3.0 (-7.0, 6.5) | 5.5  (-14.0, 15.0) | -2.0  (-21.0, 7.0) |
| 400m Walk Test: 2- Minute HR | 108.5 (97.8, 115.0) | 0.5 (-2.8, 4.0) | 107.0 (88.5, 113.5) | 1.5 (-2.2, 6.0) | 1.0  (-7.0, 13.0) | 99.0 (91.8, 105.5) | 0.0 (-19.0, 5.0) | -0.5  (-27.0, 7.0) | -1.5  (-32.0. 7.0) |
| Repeated Sit to Stand: Time, seconds | 12.5 (9.5, 15.4) | -1.3 (-1.8, -0.4) | 9.9 (9.2, 11.4) | -0.5 (-2.3, 0.4) | 0.8  (-2.2, 2.3) | 11.2 (8.8, 12.1) | -0.4 (-2.4, 0.8) | 0.9  (-1.6, 4.2) | 0.1  (-2.9, 5.1) |
| Steep Ramp Test: Max Power, watts | 124.5 (110.8, 148.0) | 0.0 (-9.0, 3.0) | 127.0 (119.5, 138.5) | 6.5 (1.2, 15.5) | 6.5  (-1.0, 27.0) | 135.0 (115.8, 162.8) | 10.0 (5.8, 12.0) | 10.0  (2.0, 34.0)  *** | 3.5  (-9.0, 14.0) |
| Steep Ramp Test: RPE | 8.0 (8.0, 8.0) | 0.0 (-1.0, 1.0) | 9.0 (7.5, 9.0) | 0.0 (0.0, 0.8) | 0.0  (-2.0, 2.0) | 8.0 (8.0, 9.0) | 0.0 (0.0, 0.0) | 0.0  (-3.0, 1.0) | 0.0  -2.0, 1.0) |
| Steep Ramp Test: Max HR, bpm | 150.0 (139.2, 156.5) | -1.5 (-5.2, 3.8) | 145.0 (131.0, 155.5) | -1.5 (-4.0, 7.0) | 0.0  (-8.0, 10.0) | 136.5 (117.0, 146.2) | -2.5 (-9.8, 6.2) | -1.0  (-14.0, 10.0) | -1.0  (-13.0, 13.0) |
|  | Control | | Resistance | | | Aerobic | | | |
|  | Baseline Median (IQR) | Median Change (IQR)^a^ | Baseline Median (IQR) | Median Change (IQR)^*^ | Median Difference from Control  (95% CI) | Baseline Median (IQR) | Median Change (IQR)^*^ | Median Difference from Control (95% CI) | Median Difference from Resistance (95% CI) |
| Steep Ramp Test: 1- Minute HR | 128.0 (122.2, 137.8) | -5.5 (-11.2, 1.8) | 119.0 (113.0, 135.0) | 1.0 (-4.0, 4.5) | 6.5  (-7.0, 21.0) | 101.0 (92.5, 116.0) | -3.0 (-8.0, 3.0) | 2.5  (-14.0, 15.0) | -4.0  (-22.0, 9.0) |
| Steep Ramp Test: 2- Minute HR | 121.0 (116.5, 131.5) | -6.0 (-16.2, -2.5) | 105.0 (93.0, 115.5) | -0.5 (-4.8, 6.0) | 5.5  (-2.0, 32.0) | 89.5 (81.5, 110.8) | -4.5 (-12.8, 2.2) | 1.5  (-13.0, 23.0) | -4.0  (-22.0, 8.0) |
| Steep Ramp Test: Max BP (systolic) | 176.0 (163.0, 186.0) | 2.0 (-18.5, 5.0) | 184.0 (164.0, 199.0) | -2.5 (-13.8, 25.2) | -4.5  (-19.0, 52.0) | 162.0 (155.0, 192.0) | 10.0 (2.5, 11.5) | 8.0  (-10.0, 32.0) | 12.5  (-48.0, 25.0) |
| Steep Ramp Test: Max BP (diastolic) | 81.0 (78.5, 84.8) | -4.0 (-4.2, -1.5) | 80.0 (79.0, 82.5) | -2.5 (-5.8, 6.0) | 1.5  (-6.0, 12.0) | 83.0 (78.0, 88.5) | -1.0 (-8.5, 2.0) | 3.0  (-10.0, 7.0) | 1.5  (-12.0, 8.0) |
| Chest Press: Actual 1RM, lbs.^**^ | 80.0 (55.0, 96.9) | 1.2 (-8.2, 11.2) | 80.0 (70.0, 82.5) | 15.0 (10.0, 17.5) | 13.8  (-12.5, 32.5) | 90.0 (62.5, 120.0) | 0.0 (0.0, 3.8) | -1.2  (-25.0, 15.0) | -15.0  (-40.0, 10.0) |
| Seated Row: Actual 1RM, lbs.^**^ | 90.0 (76.2, 116.9) | 5.0 (0.0, 8.1) | 110.0 (100.0, 120.0) | 20.0 (20.0, 30.0) | 15.0  (10.0, 30.0)  *** | 95.0 (82.5, 122.5) | 5.0 (0.0, 5.0) | 0.0  (-10.0, 35.0) | -15.0  (-35.0, 25.0) |
| Leg Press: Actual 1RM, lbs.^**^ | 210.0 (130.0, 225.0) | -17.5 (-31.2, 27.5) | 207.5 (192.5, 226.2) | 77.5 (62.5, 88.8) | 95.0  (-85.0, 135.0) | 245.0 (220.0, 325.0) | 15.0 (15.0, 35.0) | 32.5  (-110.0, 90.0), | -62.5  (-85.0, 15.0) |

Abbreviations: HR, heart rate; RPE, Rated Perceived Exertion; BP, blood pressure.

***** Baseline median calculations included all men. Median change was calculated only for men who completed both baseline and 12-week sessions. Difference is the difference in the medians.

**4 men (2 control, 1 resistance, 1 aerobic) were excluded from these calculations due to an equipment change in our exercise facility between their pre- and post-exercise visits.

***p<0.05.

| **Supporting File 5:** Estimated effect of aerobic and resistance exercise on Halabi Score (HS) and components among men with mCRPC | | | | | | | | | | | | |
| --- | --- | --- | --- | --- | --- | --- | --- | --- | --- | --- | --- | --- |
|  | Control (n=10) | | | | Resistance (n=7) | | | | Aerobic (n=8)^*^ | | | |
| Categorical HS Variables | Baseline N (%) | 12 wk. N (%) | Increase  N (%) | Decrease N (%) | Baseline N (%) | 12 wk. N (%) | Increase  N (%) | Decrease N (%) | Baseline N (%) | 12 wk. N (%) | Increase  N (%) | Decrease N (%) |
| Halabi Nomogram Score |  |  |  |  |  |  |  |  |  |  |  |  |
| Low | 8 (80) | 6 (60) | 3 (30) | 2 (20) | 5 (71) | 4 (57) | 2 (29) | 0 (0) | 5 (63) | 6 (75) | 0 (0) | 1 (13) |
| Medium | 1 (10) | 4 (40) |  |  | 2 (29) | 2 (29) |  |  | 3 (38) | 2 (25) |  |  |
| High | 1 (10) | 0 (0) |  |  | 0 (0) | 1 (14) |  |  | 0 (0) | 0 (0) |  |  |
| Disease Site |  |  |  |  |  |  |  |  |  |  |  |  |
| Lymph Node | 4 (40) | 4 (40) | 0 (0) |  | 4 (57) | 5 (71) | 1 (13) |  | 6 (75) | 6 (75) | 0 (0) |  |
| Bone | 6 (60) | 7 (70) | 1 (10) |  | 5 (71) | 5 (71) | 0 (0) |  | 6 (75) | 6 (75) | 0 (0) |  |
| Visceral | 1 (10) | 1 (10) | 0 (0) |  | 0 (0) | 0 (0) | 0 (0) |  | 0 (0) | 1 (11) | 0 (0) |  |
| ECOG Performance Status |  |  |  |  |  |  |  |  |  |  |  |  |
| 0 | 8 (80) | 6 (60) | 2 (20) |  | 4 (57) | 3 (43) |  |  | 5 (63) | 5 (63) |  |  |
| 1 | 2 (20) | 4 (40) |  | 0 (0) | 3 (43) | 4 (57) | 1 (14) | 0 (0) | 3 (38) | 3 (38) | 1 (13) | 1 (13) |
| 2 | 0 (0) | 0 (0) |  |  | 0 (0) | 0 (0) |  |  | 0 (0) | 0 (0) |  |  |
| Dichotomous HS Variables |  | | | | | | | | | | | |
| Opioid Analgesic Use | 1 (10) | 1 (10) | 0 (0) | 0 (0) | 0 (0) | 0 (0) | 0 (0) | 0 (0) | 2 (25) | 2 (25) | 0 (0) | 0 (0) |
| LDH>ULN | 0 (0) | 2 (22)* | 2 (22)* | 0 (0) | 0 (0) | 1 (14) | 1 (14) | 0 (0) | 0 (0) | 0 (0) | 0 (0) | 0 (0) |

|  | Control (n=10) | | Resistance (n=7) | | | Aerobic (n=8)^a^ | | | |
| --- | --- | --- | --- | --- | --- | --- | --- | --- | --- |
| Continuous HS Variables | Baseline Median (IQR) | Median Change (IQR)^a^ | Baseline Median (IQR) | Median Change (IQR)^a^ | Median Difference from Control  (95% CI) | Baseline Median (IQR) | Median Change (IQR)^*^ | Median Difference from Control (95% CI) | Median Difference from Resistance (95% CI) |
| Albumin, g/dL | 4.2 (3.9, 4.3) | -0.1 (-0.1, 0.2) | 3.8 (3.7, 4.0) | 0.0 (-0.1, 0.1) | 0.1  (-0.3, 0.1) | 4.1 (3.9, 4.2) | 0.0 (-0.3, 0.1) | 0.1  (-0.4, 0.2) | 0.0  (-0.4, 0.2) |
| Hemoglobin, g/dL | 13.1 (12.3, 14.6) | 0.2 (-0.5, 0.9) | 12.5 (11.8, 13.2) | 0.1 (-0.1, 0.4) | -0.1  (-1.0, 0.7) | 12.5 (12.0, 13.1) | 0.0 (-0.3, 0.1) | -0.2  (-1.2, 0.5) | -0.1  (-0.8, 0.3) |
| ALP, U/L | 73.0 (61.0, 95.3) | 0.0 (-7.0, 10.0) | 64.0 (62.5, 77.5) | 4.0 (-3.0, 9.5) | 4.0  (-15.0, 19.0) | 76.0 (52.8, 92.3) | -3.5 (-13.5, 6.5) | -3.5  (-31.0, 20.0) | -7.5  (-25.0, 13.0) |
| PSA, ng/mL | 11.1 (2.1, 32.7) | 1.1 (-0.3, 1.9) | 3.5 (1.8, 7.7) | 0.3 (0.0, 2.6) | -0.8  (-2.7, 11.7) | 1.6 (0.8, 12.4) | 0.7 (-0.4, 8.3) | -0.4  (-2.5, 37.0) | 0.4  (-4.3, 29.2) |

Abbreviations: ALP, Alkaline phosphatase; LDH, Lactate dehydrogenase; ULN, Upper normal limit; ECOG, Eastern Cooperative Oncology Group; PSA, prostate specific antigen.

*One participant did not have 12-week values.
